# NAD^+^ signaling in Parkinson's disease: molecular mechanisms and therapeutic perspectives

**DOI:** 10.3389/fneur.2026.1943801

**Published:** 2026-09-30

**Authors:** Tianbao Wang, Xue Zhou, Xinhua Chen

**Affiliations:** 1 Tianbao Hospital, Mei Hekou, China; 2 Xinjiang Medical University, Urumqi, China; 3 The Affiliated Hospital to Changchun University of Chinese Medicine, Changchun, China

**Keywords:** NAD^+^, Parkinson's disease, mitochondrial quality control, nicotinamide riboside, neuroprotection

## Abstract

Parkinson's disease (PD) is the second most prevalent neurodegenerative disorder worldwide, characterized by the progressive loss of dopaminergic neurons in the substantia nigra, accumulation of α-synuclein (α-syn), mitochondrial dysfunction, and neuroinflammation. Nicotinamide adenine dinucleotide (NAD^+^), a crucial coenzyme involved in cellular energy metabolism, DNA repair, and epigenetic regulation, declines with aging and is markedly reduced in the brains of patients with PD. Emerging evidence suggests that supplementation with NAD^+^ precursors, including nicotinamide riboside (NR) and nicotinamide mononucleotide (NMN), exerts neuroprotective effects through multiple mechanisms, such as activation of the mitochondrial unfolded protein response (UPR∧mt), enhancement of autophagy, and suppression of excessive poly(ADP-ribose) polymerase 1 (PARP1) activation. This review summarizes the central role of NAD^+^ dysregulation in the pathogenesis of PD, with particular emphasis on the interplay between NAD^+^ depletion, mitochondrial quality control, proteostasis, and neuroinflammation. Furthermore, we discuss recent advances in NAD^+^-boosting strategies in PD models and clinical studies, highlighting their potential therapeutic implications for PD intervention.

## 1 Introduction

Parkinson's disease (PD) is the second most prevalent neurodegenerative disorder, and its global burden has continued to increase with the acceleration of population aging. Current therapeutic strategies primarily rely on dopamine replacement therapy, which can alleviate motor symptoms but fails to halt or slow disease progression. Therefore, the identification of disease-modifying therapeutic approaches has become an urgent priority in the field.

Nicotinamide adenine dinucleotide (NAD^+^) and its phosphorylated form, nicotinamide adenine dinucleotide phosphate (NADP^+^), play fundamental roles in all living organisms. Through interconversion with their reduced forms, NADH and NADPH, they function as carriers of reducing equivalents during anabolic and catabolic processes and participate in numerous energy-generating biochemical reactions. These processes include the biosynthesis of lipids, nucleic acids, and steroid hormones, as well as the maintenance of cellular redox balance and protection against oxidative stress. In addition to its classical role as a redox coenzyme, NAD^+^ serves as a critical signaling co-substrate for several enzyme families, including sirtuin deacetylases, poly(ADP-ribose) polymerases (PARPs), cyclic ADP-ribose (cADPR) synthases, and sterile alpha and TIR motif-containing protein 1 (SARM1). These NAD^+^-dependent enzymes are involved in diverse biological processes, including metabolic regulation, DNA repair, and aging-related pathways. In particular, NAD^+^ acts not only as a central mediator of cellular redox reactions but also as a substrate for key regulatory enzymes, such as the Sirtuin and PARP families, thereby modulating mitochondrial function, DNA repair, inflammatory responses, and autophagy.

Recent studies have revealed that disruption of NAD^+^ homeostasis may represent a critical link between aging and PD pathogenesis. A significant reduction in NAD^+^ levels has been observed in both brain tissues from patients with PD and experimental PD models, providing a theoretical basis for therapeutic strategies targeting NAD^+^ metabolism. In this review, we summarize the role of NAD^+^ in PD pathogenesis from three perspectives: underlying molecular mechanisms, preclinical evidence, and clinical translation, and discuss recent advances in NAD^+^-based therapeutic approaches.

## 2 Mechanisms of NAD^+^ biosynthesis

In mammalian cells, NAD^+^ can be synthesized from multiple metabolic intermediates and precursor molecules through distinct biosynthetic pathways. Vitamin B3, a group of water-soluble vitamins, includes nicotinic acid (NA) and nicotinamide (NAM), both of which serve as important precursors for NAD^+^ biosynthesis (1). These precursors contribute to NAD^+^ production through three major pathways: the *de novo* pathway, the Preiss–Handler pathway, and the salvage pathway.

### 2.1 *De novo* pathway

The *de novo* pathway utilizes tryptophan as the initial substrate and represents the only NAD^+^ biosynthetic route in mammals that can generate NAD^+^ from non-NAD-related precursors. This pathway is also known as the kynurenine pathway (KP) and involves multiple enzymatic reactions, occurring predominantly in the liver, immune cells, and the brain. Tryptophan is initially converted into N-formylkynurenine through the activity of tryptophan 2,3-dioxygenase (TDO) in the liver or indoleamine 2,3-dioxygenase 1/2 (IDO1/2) in immune cells and the brain. N-formylkynurenine is subsequently converted into kynurenine (KYN) following deformylation by kynurenine formamidase, which represents a critical branching point in this pathway. KYN is then metabolized by kynurenine 3-monooxygenase (KMO) to generate 3-hydroxykynurenine (3-HK), followed by a series of enzymatic reactions leading to the formation of quinolinic acid (QA). QA is converted into nicotinic acid mononucleotide (NAMN) by quinolinate phosphoribosyltransferase (QPRT), which subsequently enters the downstream steps of the Preiss–Handler pathway and is ultimately converted into NAD^+^ (2).

Compared with other NAD^+^ biosynthetic pathways, the *de novo* pathway is relatively complex and inefficient, and its activity is tightly regulated by inflammatory signals. For example, IDO1 expression can be induced by pro-inflammatory cytokines such as interferon-γ (IFN-γ). Moreover, metabolic intermediates within the KP can be diverted toward alternative pathways. When KYN is converted into kynurenic acid (KA) by kynurenine aminotransferases (KYATs), NAD^+^ biosynthesis is interrupted. Although enzymes involved in *de novo* NAD^+^ synthesis are expressed in the central nervous system (CNS), their distribution varies among different cell types. Microglia express most, if not all, of the enzymes required for *de novo* NAD^+^ synthesis, whereas such expression has not been clearly detected in astrocytes. However, astrocytes can produce substantial amounts of QA under certain conditions. Neurons express IDO1, but appear to lack sufficient enzymatic capacity to convert tryptophan into NAD^+^ efficiently, which may be attributed to the potential neurotoxicity of several intermediates generated during this pathway.

### 2.2 Preiss–Handler pathway

The Preiss–Handler pathway is the first identified NAD^+^ biosynthetic pathway and utilizes nicotinic acid (NA) as the precursor. This pathway directly converts exogenous NA into NAD^+^ and is mainly active in tissues such as the liver, kidney, and intestine, whereas high expression of this pathway has not been observed in the CNS (3). NA is first converted into nicotinic acid mononucleotide (NAMN) by nicotinic acid phosphoribosyltransferase (NAPRT). NAMN is subsequently converted into nicotinic acid adenine dinucleotide (NAAD) by nicotinamide mononucleotide adenylyltransferases (NMNAT1–3). Finally, NAAD undergoes amidation catalyzed by NAD synthetase (NADSYN) to generate NAD^+^. Compared with the *de novo* pathway, the Preiss–Handler pathway is relatively direct and moderately efficient, and is less affected by feedback inhibition from NAD^+^ metabolites.

### 2.3 Salvage pathway

The salvage pathway utilizes NAD^+^ degradation products as substrates and represents the predominant and most efficient NAD^+^ biosynthetic pathway in mammals (4). Approximately 80% of cellular NAD^+^ production is attributed to this pathway. It recycles NAD^+^ breakdown products and is widely distributed across tissues, with particularly high activity in organs with substantial energy demands, including the brain and skeletal muscle. The major precursors involved in the salvage pathway include nicotinamide (NAM), nicotinamide riboside (NR), and nicotinamide mononucleotide (NMN). Nicotinamide phosphoribosyltransferase (NAMPT) is a key metabolic enzyme that plays an essential role in NAD^+^ biosynthesis. As a critical regulator of cellular NAD^+^ availability, NAMPT contributes to redox regulation, energy metabolism, and NAD^+^-dependent signaling processes (5). NAM is converted into NMN by NAMPT, representing the rate-limiting step of the salvage pathway (6). Subsequently, NMN is converted into NAD^+^ by nicotinamide mononucleotide adenylyltransferases (NMNAT1–3) (7). Three NMNAT isoforms (NMNAT1–3) have been identified, each exhibiting distinct subcellular localization patterns. NMNAT1 is primarily localized in the nucleus, NMNAT2 is present in the cytoplasm and Golgi apparatus, and NMNAT3 is mainly found in mitochondria and the cytoplasm (8). Among these isoforms, NMNAT2 is highly expressed in the CNS and plays essential roles in axonal survival and synaptic function (9, 10). In addition to the NAMPT-dependent route, NMN can also be generated through an alternative NR-dependent pathway. Nicotinamide riboside (NR) is phosphorylated by nicotinamide riboside kinases (NRK1/2) to produce NMN, which subsequently enters the NMNAT-mediated step for NAD^+^ synthesis (11). Although exogenously supplemented NMN can theoretically be directly converted into NAD^+^ by NMNAT1–3, its oral absorption in humans may depend on intestinal microbiota-mediated metabolism or conversion into NR before cellular uptake. The salvage pathway is characterized by rapid synthesis, low energy consumption, and high efficiency, making it the central mechanism responsible for maintaining intracellular NAD^+^ homeostasis.

## 3 Biological functions of NAD^+^

The biological functions of NAD^+^ encompass fundamental cellular processes, including metabolism, signal regulation, and stress responses, making it an essential molecule for maintaining cellular activities and organismal homeostasis. Within cells, NAD^+^ exists primarily in two interconvertible forms: the oxidized form (NAD^+^) and the reduced form (NADH). Its major biological functions can be broadly categorized into two aspects: serving as an electron carrier in energy metabolism and acting as a consumable substrate in signal transduction pathways.

### 3.1 NAD^+^ as a mediator of energy metabolism

As an essential electron carrier, NAD^+^ participates in several fundamental metabolic pathways, including glycolysis, the tricarboxylic acid (TCA) cycle, and oxidative phosphorylation. Through reversible redox reactions, NAD^+^ accepts electrons and is converted into its reduced form, NADH, which subsequently transfers electrons to the mitochondrial respiratory chain to drive ATP production. During glycolysis, glyceraldehyde-3-phosphate dehydrogenase catalyzes the oxidation of glyceraldehyde-3-phosphate, transferring electrons to NAD^+^ to generate NADH. NADH is then transported into mitochondria, where it donates electrons to complex I of the respiratory chain, ultimately promoting oxidative phosphorylation and ATP synthesis.

The NAD^+^/NADH ratio serves as a molecular indicator of the cellular redox state and directly regulates the activity of key metabolic enzymes, including pyruvate dehydrogenase (PDH) and lactate dehydrogenase (LDH). An increased NAD^+^/NADH ratio reflects a more oxidized intracellular environment, favoring aerobic metabolism and fatty acid synthesis, whereas a decreased ratio indicates a more reduced state, promoting glycolysis and lactate production to prevent excessive oxidative stress. In PD, mitochondrial dysfunction impairs the efficient regeneration of NAD^+^ from NADH, resulting in a reduced NAD^+^/NADH ratio, which contributes to metabolic failure and reactive oxygen species (ROS) accumulation.

In addition to its role as an electron acceptor, NAD^+^ can be phosphorylated by NAD^+^ kinases (NADKs) to generate NADP^+^, while NAD^+^/NADH redox cycling is mediated by various dehydrogenases. The NAD^+^/NADH redox couple is considered a key regulator of cellular energy metabolism, particularly in coordinating glycolysis and mitochondrial oxidative phosphorylation. In contrast, NADP^+^ and its reduced form NADPH primarily contribute to the maintenance of cellular redox homeostasis and support biosynthetic processes, including fatty acid and nucleotide synthesis.

### 3.2 NAD^+^ involvement in signal transduction

#### 3.2.1 NAD^+^-dependent ADP-ribosylation regulates mitochondrial function

ADP-ribosylation is a post-translational modification (PTM) that uses NAD^+^ as the direct substrate. Among its various forms, poly(ADP-ribosylation; PARylation) is the most extensively studied modification. PARP family enzymes utilize NAD^+^ as an ADP-ribose donor, transferring ADP-ribose moieties to target proteins and generating poly(ADP-ribose; PAR) chains, thereby regulating diverse biological processes, including DNA damage responses, chromatin remodeling, gene transcription, and mitochondrial function. In addition to PARPs, ADP-ribosyl cyclases such as CD38 and CD157 also use NAD^+^ as a substrate to catalyze the formation of cyclic ADP-ribose (cADPR), which participates in intracellular Ca^2+^ signaling and immune regulation.

Recent studies have suggested that PARylation may also occur within mitochondria; however, the existence and biological functions of mitochondrial PARP (mtPARP) remain controversial. Traditionally, PARP1 has been considered a predominantly nuclear enzyme involved in DNA damage repair. However, under certain stress conditions, such as oxidative stress, PARP1 can translocate to mitochondria and interact with mitochondrial proteins, including mitochondrial DNA polymerase γ (POLG). Using isolated mitochondrial systems, researchers have demonstrated that exogenous NAD^+^ markedly enhances mitochondrial PARylation, whereas the PARP1 inhibitor olaparib significantly suppresses this process. Furthermore, approaches including end-labeling of ADP-ribose (ELTA), ADP-ribose chromatin affinity purification (ADPr-ChAP), and PARP1 chromatin immunoprecipitation (ChIP) have provided evidence that NAD^+^ promotes PARylation of the mitochondrial DNA control region known as the displacement loop (D-loop). This modification enhances the binding of mitochondrial transcription factor A (TFAM) to the D-loop region, thereby facilitating mitochondrial DNA (mtDNA) transcription (12). Collectively, these findings suggest that NAD^+^-dependent PARylation may contribute to the regulation of mitochondrial gene expression and function.

#### 3.2.2 NAD^+^-dependent regulation of DNA damage repair

NAD^+^ primarily functions as a co-substrate for PARP family enzymes during the DNA damage repair process. Upon the occurrence of single-strand breaks (SSBs) or double-strand breaks (DSBs), PARP1 rapidly recognizes DNA lesions and utilizes NAD^+^ to catalyze PARylation of itself and associated target proteins. The resulting PAR chains not only serve as molecular scaffolds to recruit DNA repair factors, including X-ray repair cross-complementing protein 1 (XRCC1), DNA ligase III, and DNA polymerase β, but also facilitate multiple DNA repair pathways, such as base excision repair (BER), homologous recombination (HR), and non-homologous end joining (NHEJ), thereby maintaining genomic stability. PARP activity is tightly regulated by intracellular NAD^+^ availability. When cellular NAD^+^ levels decline, the NAD^+^-binding protein deleted in breast cancer 1 (DBC1) interacts with PARP1 to form an inhibitory complex, resulting in reduced PARP1 catalytic activity and impaired DNA repair capacity (13). A study demonstrated that mice with increased formation of the DBC1–PARP1 complex exhibited decreased PARP activity, accumulated DNA damage, and impaired HR and NHEJ repair efficiency, highlighting the essential role of NAD^+^ homeostasis in regulating DNA damage responses (14) (Figure 1).

**Figure 1:**
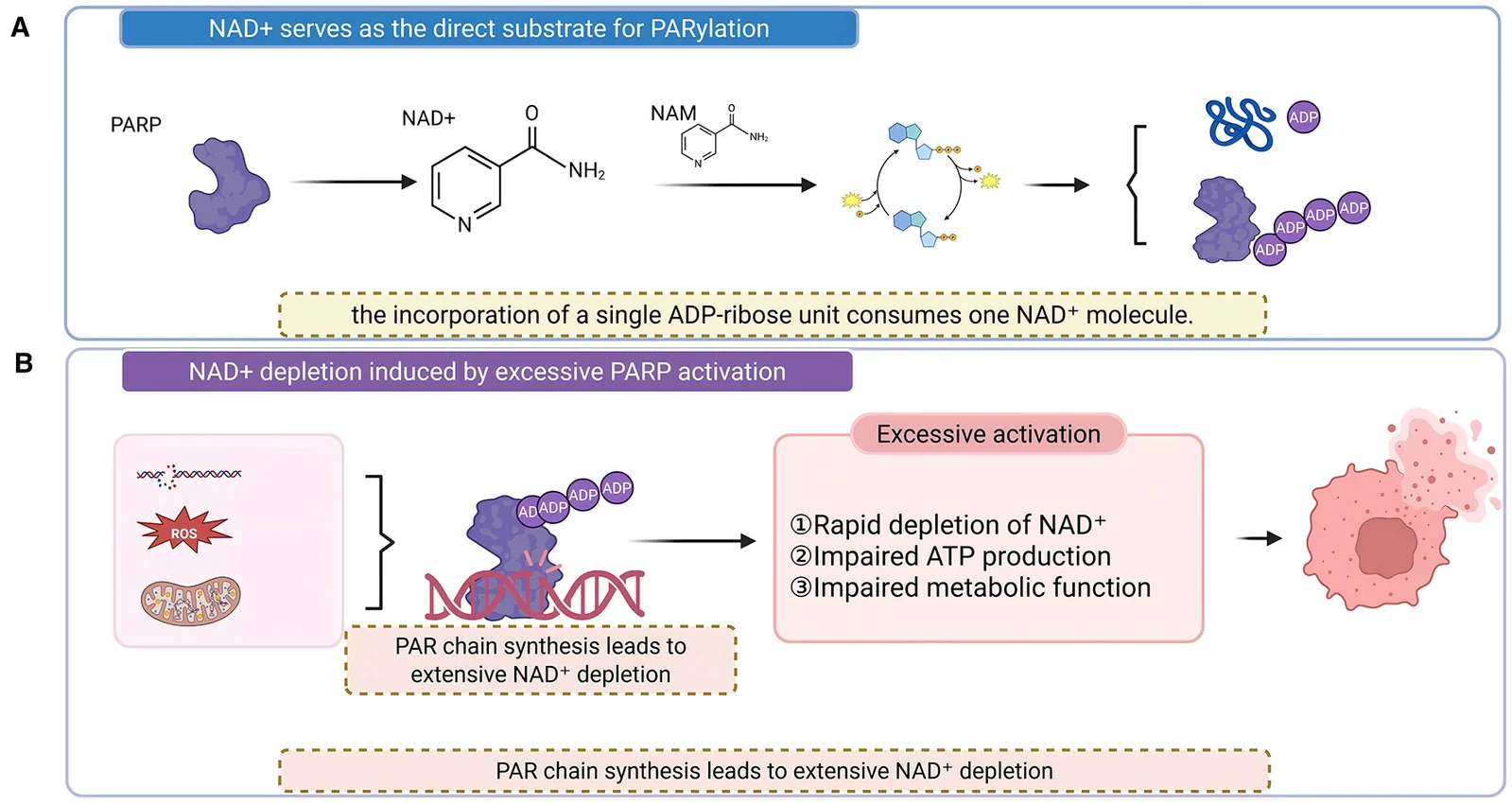
**(A)** NAD^+^ is the direct substrate for PARylation; **(B)** PARylation is an NAD^+^-consuming signaling pathway.

Although PARP1 is indispensable for maintaining genomic stability, its excessive and sustained activation can exert detrimental effects. Under conditions of persistent oxidative stress, extensive DNA damage induces prolonged PARP1 activation, leading to excessive consumption of NAD^+^ and rapid depletion of both intracellular NAD^+^ and ATP. This metabolic crisis further promotes mitochondrial dysfunction, exacerbates oxidative stress, and ultimately triggers cell death (15). Therefore, appropriate regulation of PARP activity is required to maintain a balance between DNA repair demands and cellular energy metabolism. Recent studies have demonstrated that increasing cellular NAD^+^ availability can enhance DNA repair capacity and improve mitochondrial function (16). Supplementation with NAD^+^ precursors or moderate inhibition of PARP1 can restore intracellular NAD^+^ levels, improve mitochondrial oxidative metabolism, reduce reactive oxygen species (ROS) production, and promote mitochondrial biogenesis (17). In DNA repair-deficient models, such interventions not only rescue mitochondrial dysfunction but also enhance mitochondrial DNA (mtDNA) base excision repair capacity, improve tissue function, and extend lifespan in experimental animals (18). Collectively, the NAD^+^/PARP1 axis represents a critical metabolic regulatory hub connecting DNA damage repair, mitochondrial homeostasis, and aging-related disorders, and may serve as a promising therapeutic target for neurodegenerative diseases.

#### 3.2.3 Deacetylation mediated by sirtuins

NAD^+^ is an essential coenzyme required for the catalytic activity of the silent information regulator (sirtuin, SIRT1–SIRT7) family of deacetylases. Through NAD^+^-dependent deacetylation, sirtuins regulate a wide range of substrates, including histones, transcription factors, and metabolic enzymes, thereby participating in diverse biological processes such as energy metabolism, oxidative stress responses, mitochondrial homeostasis, and cellular senescence. Among the sirtuin family members, SIRT1 activity is highly dependent on intracellular NAD^+^ availability. In the nucleus, SIRT1 promotes mitochondrial biogenesis by deacetylating peroxisome proliferator-activated receptor gamma coactivator 1-alpha (PGC-1α), a master regulator of mitochondrial biogenesis. Activated PGC-1α subsequently induces the expression of nuclear respiratory factor 1 (NRF1) and mitochondrial transcription factor A (TFAM), facilitating mitochondrial DNA (mtDNA) replication and transcription, as well as the expression of respiratory chain-related proteins, thereby enhancing mitochondrial function (19). In addition, SIRT1-mediated deacetylation of forkhead box O3a (FOXO3a) promotes the expression of antioxidant genes, including superoxide dismutase 2 (SOD2), and activates autophagy-related pathways, thereby improving cellular resistance to oxidative stress (20). A decline in intracellular NAD^+^ levels results in reduced SIRT1 activity, leading to impaired PGC-1α-dependent mitochondrial biogenesis and weakened FOXO3a-mediated antioxidant responses. Within the NAD^+^ salvage pathway, NR and NMN serve as key precursors, and their metabolic utilization is regulated by NAD^+^ availability and the activity of related enzymes. Therefore, disruption of NAD^+^ homeostasis may indirectly alter the metabolic flux through the salvage pathway.

SIRT3, a major mitochondrial matrix-localized deacetylase, plays a critical role in maintaining mitochondrial homeostasis by regulating key enzymes involved in the tricarboxylic acid (TCA) cycle, electron transport chain complexes, and antioxidant defense systems. In particular, SIRT3 is essential for preserving mitochondrial integrity under oxidative stress conditions. Through modulation of mitochondrial protein acetylation status, SIRT3 contributes to the maintenance of mitochondrial proteostasis, regulation of mitochondrial dynamics, and control of mitochondrial autophagy (mitophagy). Under conditions of mitochondrial dysfunction, SIRT3 has been implicated in the regulation of PINK1/PARKIN-dependent mitophagy, thereby facilitating the removal of damaged mitochondria (21). Furthermore, SIRT3-mediated deacetylation of mitochondrial respiratory chain complexes, including complex I and complex II, as well as manganese superoxide dismutase (MnSOD), enhances oxidative phosphorylation efficiency and maintains cellular redox homeostasis.

SIRT6 is predominantly localized in the nucleus and contributes to genomic stability, DNA damage repair, and regulation of glucose and lipid metabolism, playing an important role in aging-related processes. Collectively, NAD^+^ serves not only as an indispensable coenzyme for sirtuin-mediated deacetylation but also as a central metabolic hub connecting mitochondrial biogenesis, oxidative stress responses, mitochondrial quality control, and neuroprotective mechanisms. Consequently, impaired NAD^+^ homeostasis may represent a critical pathological factor in neurodegenerative disorders, including Parkinson's disease (Figure 2).

**Figure 2:**
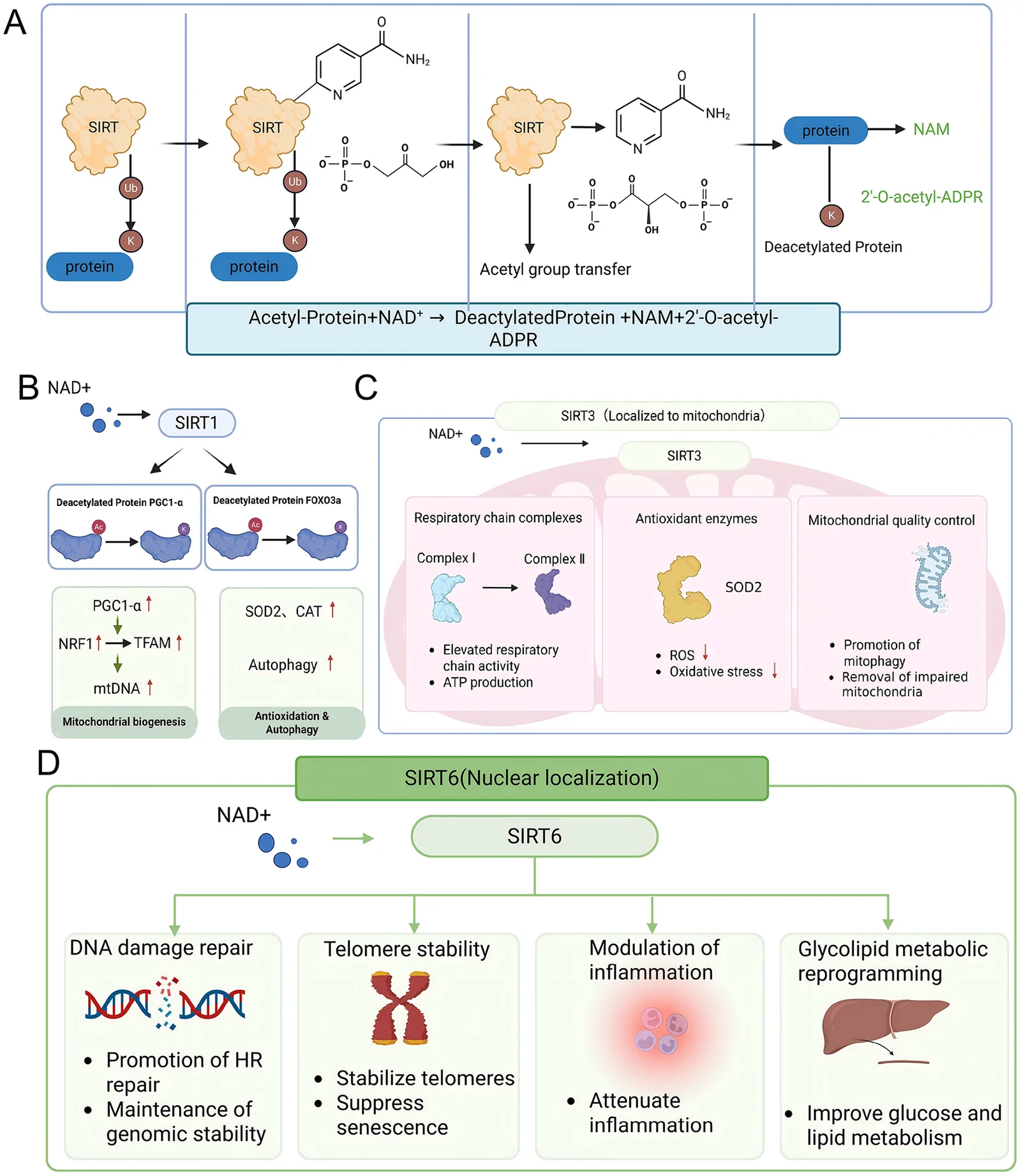
**(A)** Mechanism of SIRT deacetylation: direct involvement of NAD^+^ as a co-substrate; **(B–D)** Deacetylation targets and biological effects of major SIRT family members. ROS, reactive oxygen species; NRF1, nuclear respiratory factor 1; TFAM, mitochondrial transcription factor A; SOD2, superoxide dismutase 2; CAT, Catalase.

#### 3.2.4 Regulation of autophagy

Autophagy is an evolutionarily conserved catabolic process that maintains cellular homeostasis and facilitates adaptation to environmental stress by degrading and recycling damaged or excessive proteins, organelles, and other macromolecular components. Increasing evidence indicates that autophagy and NAD^+^ homeostasis are closely interconnected through a bidirectional regulatory relationship (22). Mechanistically, NAD^+^-dependent enzymes, including sirtuins and PARPs, can directly regulate autophagy and mitochondrial quality control. Conversely, autophagic degradation and recycling of metabolic intermediates and NAD^+^ precursors, such as NAM, contribute to the maintenance of intracellular NAD^+^ homeostasis (23). In addition, several NAD^+^-dependent enzymes participate in the regulation of autophagosome formation, lysosomal function, and mitophagy.

Studies using autophagy-deficient models have demonstrated that loss of autophagic activity disrupts cellular metabolic balance and NAD^+^ homeostasis, leading to mitochondrial dysfunction, impaired proteostasis, and increased cell death (24, 25). The underlying mechanisms may involve enhanced DNA damage and inflammatory responses, which activate NAD^+^-consuming enzymes, including PARPs, CD38/CD157, and SARM1, thereby further depleting intracellular NAD^+^ levels. Meanwhile, NAD^+^ depletion suppresses the activity of sirtuin family enzymes, establishing a vicious cycle of metabolic dysregulation and cellular injury (22, 26). Within the central nervous system (CNS), SIRT1, SIRT2, and SIRT3 represent key effectors involved in NAD^+^-mediated regulation of autophagy. Among them, SIRT1 is predominantly localized in the nucleus and promotes autophagy and mitochondrial biogenesis through deacetylation of FOXO3a and PGC-1α. SIRT1 also regulates autophagic processes by modulating autophagy-related proteins, including members of the ATG family and LC3-associated pathways (27). SIRT1 deficiency results in impaired autophagy and disrupted energy homeostasis, whereas SIRT1 overexpression can induce autophagy even under nutrient-rich conditions (28). Further studies have demonstrated that NAD^+^ activates the SIRT1/FOXO3a signaling pathway, leading to increased expression of autophagy-related genes, including ATG5, ATG7, and LC3, and promotes autophagy initiation and mitophagy through the SIRT1–AMPK–ULK1 signaling axis (29). Recent studies have further revealed that SIRT3 facilitates the clearance of damaged mitochondria by regulating PINK1/PARKIN-dependent mitophagy. SIRT3 overexpression has been shown to alleviate mitochondrial dysfunction and oxidative stress-induced damage in models of neurological disorders (30).

In addition, age-associated NAD^+^ decline has been observed in both aged mice and Caenorhabditis elegans. Artificial reduction of NAD^+^ levels in C. elegans further shortens lifespan, whereas genetic or pharmacological restoration of NAD^+^ availability ameliorates age-related metabolic decline and extends lifespan. These beneficial effects involve *sir-2.1*, the *Caenorhabditis elegans* ortholog of mammalian SIRT1, suggesting that SIRT1-like signaling contributes to NAD^+^-mediated longevity and stress adaptation. The underlying mechanisms involve induction of the mitochondrial–nuclear protein homeostasis response, activation of the mitochondrial unfolded protein response (UPR∧mt), and promotion of nuclear translocation of the FOXO transcription factor DAF-16 (31, 32). These pathways collectively induce the expression of autophagy-related genes, thereby enhancing cellular stress adaptation and survival capacity. In mammalian cells, SIRT1 can additionally promote autophagosome formation by deacetylating Beclin-1 and facilitating its dissociation from the Bcl-2 complex, thereby enhancing autophagic activity (33).

Collectively, autophagy and NAD^+^ form an interdependent regulatory feedback network that jointly maintains cellular metabolic homeostasis, mitochondrial quality control, and neuronal survival. Dysregulation of this network may contribute to the initiation and progression of neurodegenerative disorders, including Parkinson's disease.

#### 3.2.5 Involvement in apoptosis regulation

It is noteworthy that depletion of NAD^+^ and NADH can trigger a cytotoxic cascade involving mitochondrial depolarization and oxidative stress, ultimately leading to apoptotic cell death. Under conditions of NAD^+^ deficiency, excessive activation of PARP1 results in further NAD^+^ depletion and ATP reduction, thereby inducing caspase-dependent apoptosis. In contrast, SIRT1 can suppress neuronal apoptosis by deacetylating p53, a key pro-apoptotic protein, and inhibiting its transcriptional activity (23).

#### 3.2.6 Involvement in epigenetic regulation

NAD^+^ is closely linked to epigenetic regulation through multiple mechanisms. Although NAD^+^ itself does not directly serve as a methyl donor for DNA methylation, its metabolic status is tightly coupled with the one-carbon metabolism network and can indirectly influence the synthesis and utilization of S-adenosylmethionine (SAM). SAM serves as the primary methyl donor for DNA methyltransferases (DNMTs)-mediated DNA methylation reactions. Therefore, alterations in NAD^+^ homeostasis can reshape epigenetic states through metabolic network remodeling (34). In addition, NAD^+^ participates in epigenetic regulation through NAD^+^-dependent enzyme families, including sirtuins (SIRTs) and poly(ADP-ribose) polymerases (PARPs). SIRTs regulate chromatin accessibility and gene transcription by catalyzing the deacetylation of histones and non-histone substrates, whereas PARPs modulate chromatin architecture, DNA damage responses, and DNA methylation-related processes through ADP-ribosylation. Recent studies have further suggested that NAD^+^ may exert relatively independent epigenetic regulatory effects. Age-related or nutrition-associated NAD^+^ decline is closely associated with the establishment of aberrant DNA methylation patterns at specific genomic loci. *In vitro* studies have demonstrated that PAR polymers generated by PARP1 can inhibit the activity of DNA methyltransferase 1 (DNMT1), thereby influencing DNA methylation status (35). Moreover, ADP-ribosylation modifications can regulate the transcriptional activity of genes such as P16 and TET1 (36). For example, NAD^+^ supplementation promotes PAR polymer formation, which induces site-specific demethylation of the distal promoter region of the transcription factor CCAAT/enhancer-binding protein alpha (CEBPA). This process significantly enhances CEBPA mRNA expression in K562 and HEK293 cells (37). However, this effect appears to be highly dependent on cellular context. In Jurkat cells, a T-cell line characterized by extensive methylation of the CEBPA locus, NAD^+^ treatment resulted in only a modest increase in CEBPA expression, with approximately a 20% reduction in DNA methylation levels at this locus after 72 h. These findings indicate that NAD^+^-mediated epigenetic remodeling is influenced by cellular background and pre-existing methylation patterns (38).

Another study demonstrated that reducing NAD^+^ levels in primary mouse cortical neurons suppresses neuronal activity-dependent expression of brain-derived neurotrophic factor (BDNF), independent of SIRT or PARP activity. Mechanistically, NAD^+^ deficiency promotes increased DNA methylation within the BDNF promoter region, leading to the dissociation of CCCTC-binding factor (CTCF) and its associated cohesin complex from this locus. These alterations in higher-order chromatin organization further modify local histone acetylation and methylation patterns, ultimately suppressing BDNF transcription (39). This study suggests that, beyond canonical SIRT- and PARP-dependent pathways, NAD^+^ may also regulate gene expression through modulation of DNA methylation and higher-order chromatin architecture.

Collectively, NAD^+^ functions not only as a central metabolic coenzyme but also as a metabolic hub connecting multiple epigenetic mechanisms, including DNA methylation, histone modification, ADP-ribosylation, and chromatin remodeling. Dysregulation of NAD^+^ homeostasis may contribute to aging and neurodegenerative disease progression through epigenetic reprogramming (Figure 3).

**Figure 3:**
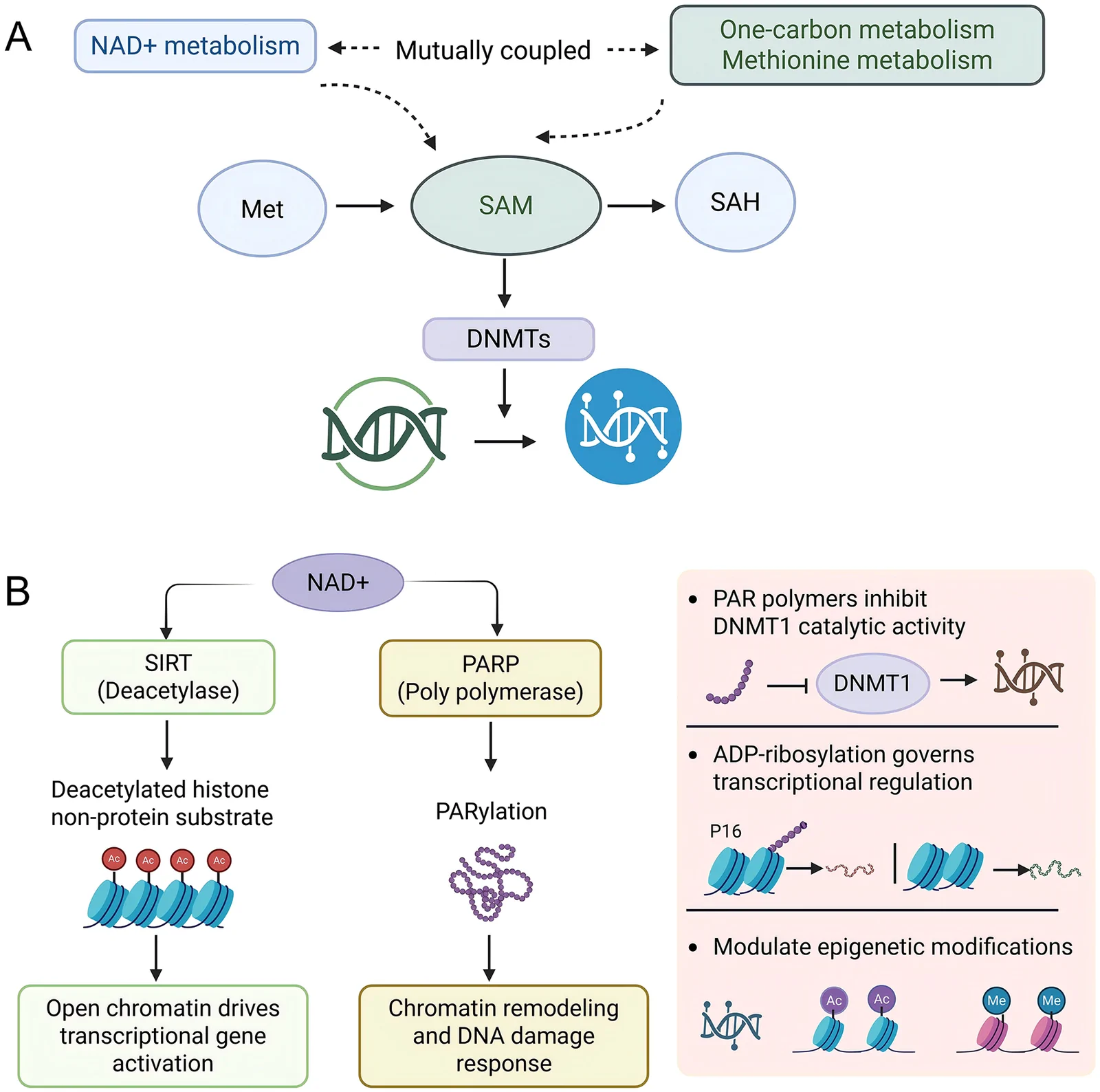
**(A)** Coupling of NAD^+^ and methyl donor metabolism; **(B)** NAD^+^ as a substrate for SIRT and PARP family enzymes regulates chromatin and DNA methylation.

#### 3.2.7 Regulation of immune responses and inflammation

NAD^+^ plays an important role in regulating immune cell function and inflammatory responses. As both a critical metabolic coenzyme and signaling molecule, NAD^+^ influences immune cell activation, cytokine production, and inflammatory signaling pathways through the regulation of key molecules, including CD38, sirtuins, and PARPs.

CD38 is a transmembrane glycoprotein widely expressed on the surface of immune cells, including T cells, B cells, macrophages, and microglia, and also functions as a major NAD^+^-consuming enzyme. By catalyzing the conversion of NAD^+^ into cyclic ADP-ribose (cADPR) and other metabolites, CD38 contributes to intracellular calcium mobilization and immune cell activation. Increased CD38 activity leads to intracellular NAD^+^ depletion, thereby disrupting metabolic homeostasis and immune function. Studies have demonstrated that inhibition of CD38 elevates intracellular NAD^+^ levels and alleviates neuroinflammation and tissue injury (40).

Recent studies have revealed that NAD^+^ dysregulation is closely associated with activation of the cGAS–STING-mediated innate immune response. In models of neurodegenerative diseases, including Alzheimer's disease (AD), reduced intracellular NAD^+^ levels are accompanied by mitochondrial dysfunction and accumulation of cytosolic DNA, which subsequently promotes activation of the cGAS–STING signaling pathway. Studies using microglial culture systems have shown that supplementation with NR exerts neuroprotective effects by modulating the cGAS–STING pathway and reducing cytosolic DNA accumulation in APP/PS1 transgenic mice and fibroblasts derived from AD patients, thereby alleviating inflammatory responses and cellular senescence phenotypes (41).

Elevated NAD^+^ availability can also suppress the expression of multiple pro-inflammatory cytokines, including interleukin-1β (IL-1β), tumor necrosis factor-α (TNF-α), and interleukin-6 (IL-6). In a high glucose-induced mouse model, NR treatment significantly reduced IL-1β expression in brain tissue and inhibited the production of inflammatory cytokines such as TNF-α and IL-6. Furthermore, NR suppressed NLRP3 inflammasome assembly by downregulating the expression of NLRP3, ASC, and caspase-1, thereby mitigating inflammatory injury. In a chronic cerebral hypoperfusion (CCH) model, NAD^+^ supplementation improved cognitive function and reduced neuroinflammation by enhancing mitochondrial function, decreasing ROS production, and restoring energy metabolism. *In vitro* studies further demonstrated that NAD^+^ protects against hypoxia-induced mitochondrial damage and inflammatory responses, partly through activation of the SIRT1/PGC-1α pathway, which promotes mitochondrial biogenesis and enhances ROS clearance capacity (19).

Beyond directly regulating inflammatory signaling pathways, NAD^+^ also influences macrophage polarization. Increased NAD^+^ availability has been shown to suppress polarization toward the pro-inflammatory M1 macrophage phenotype while promoting the formation of the anti-inflammatory M2 phenotype. Moreover, the NAD^+^-dependent deacetylase SIRT1 suppresses inflammatory responses by deacetylating the NF-κB p65 subunit, thereby inhibiting its transcriptional activity and nuclear translocation and reducing the expression of pro-inflammatory genes.

The extracellular anti-inflammatory effects mediated by NAD^+^ are closely associated with CD73 (42). CD73 is an extracellular 5′-nucleotidase involved in regulating the conversion of NAD^+^ degradation products into adenosine (43). Adenosine generated by CD73 activates adenosine receptors and exerts potent anti-inflammatory effects, including suppression of T-cell activation and inflammatory cytokine release. Knockdown of CD73 in cultured lymphocytes induces a pro-inflammatory phenotype, whereas CD73-deficient mice exhibit enhanced microglial activation and exacerbated neuroinflammatory responses in lipopolysaccharide (LPS)-induced inflammatory models (44).

Importantly, the relationship between NAD^+^ and inflammation is not simply inversely correlated. Although NAD^+^ depletion is generally associated with chronic inflammation and tissue injury, under certain pathological conditions, enhanced NAMPT-mediated NAD^+^ salvage synthesis may provide sufficient substrate for sustained PARP1 activation, thereby exacerbating NAD^+^ and ATP consumption and promoting cellular dysfunction (45). Therefore, the role of NAD^+^ in inflammatory regulation is highly context-dependent and is determined by factors including cell type, metabolic status, and the activation state of associated signaling pathways (Figure 4).

**Figure 4:**
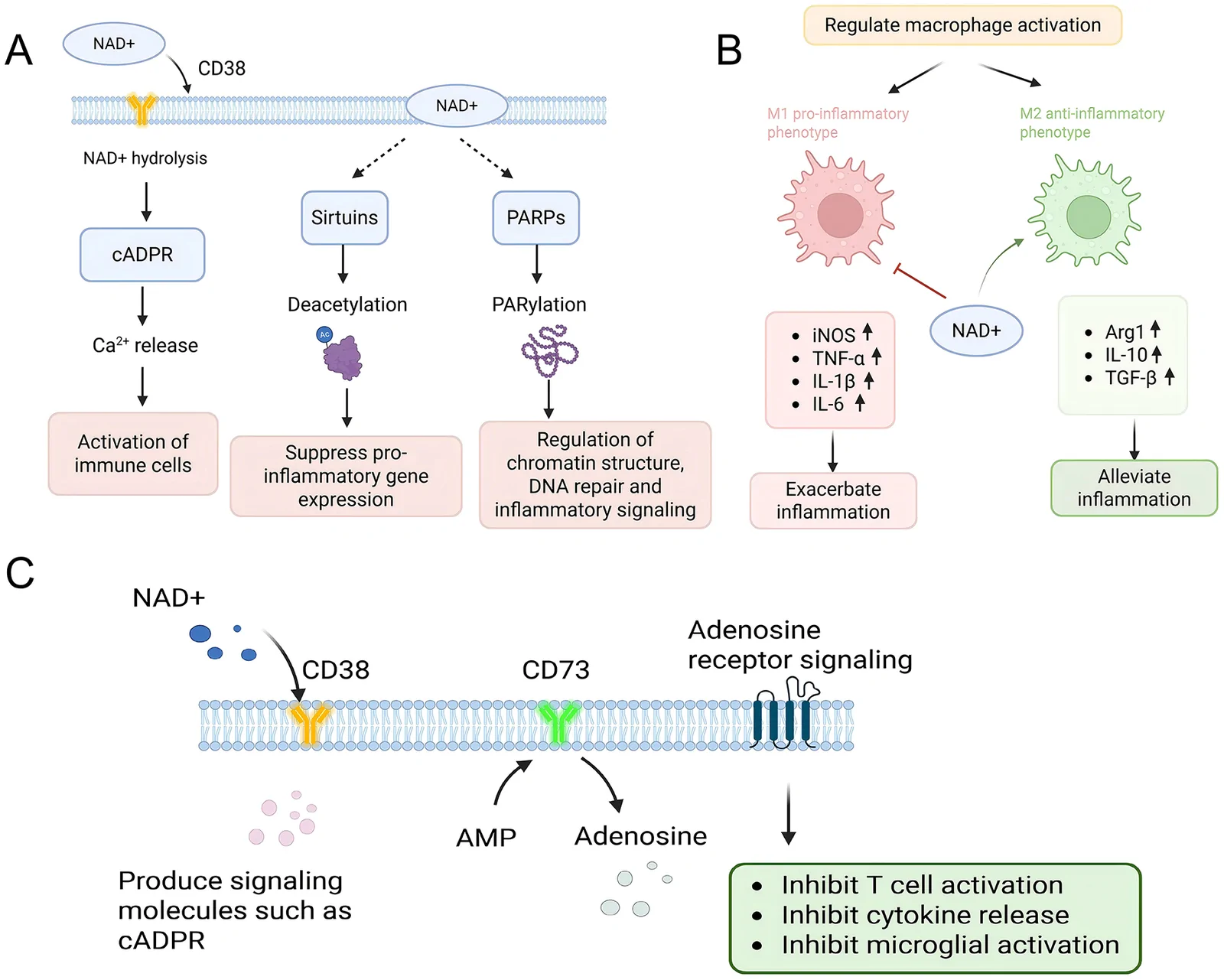
**(A)** Metabolism and signaling functions of NAD^+^ in immune cells; **(B)** NAD^+^ regulates macrophage polarization; **(C)** Anti-inflammatory effects of extracellular NAD^+^.

## 4 NAD^+^ depletion in patients with Parkinson's disease and disease models

PD is characterized by significant disturbances in NAD^+^ metabolism. Impaired mitochondrial complex I activity is one of the classical hypotheses underlying PD pathogenesis, and dysfunction of complex I directly alters the NAD^+^/NADH ratio, resulting in cellular energy failure. In addition, elevated oxidative stress under pathological conditions in PD promotes the accumulation of DNA damage, leading to excessive activation of PARP1. Hyperactivated PARP1 consumes large amounts of NAD^+^, thereby establishing a vicious cycle of NAD^+^ depletion, energy deficiency, and neuronal death. Magnetic resonance spectroscopy and imaging (MRS) have been applied to evaluate mitochondrial and cellular bioenergetic dysfunction *in vivo*, including maximal mitochondrial ATP production capacity (ATPmax) and NAD^+^ concentrations associated with energy metabolism and stress response pathways. Studies have demonstrated that ATPmax and NAD^+^ levels are significantly reduced in patients with PD. Exploratory analyses of mitochondrial biomarkers and clinical manifestations further revealed that higher ATPmax levels are associated with greater vitality and engagement, as well as a lower prevalence of rapid eye movement sleep behavior disorder (RBD) (46). Postmortem analyses of brain tissues from patients with PD have revealed alterations in the kynurenine pathway, including reduced kynurenic acid levels and decreased L-kynurenine concentrations in the frontal cortex, putamen, and substantia nigra. Peripheral blood analyses have shown more complex metabolic changes: kynurenic acid levels are elevated in erythrocytes, whereas the plasma kynurenic acid/L-kynurenine ratio is decreased and quinolinic acid levels are increased. Cerebrospinal fluid (CSF) analyses consistently demonstrate reduced kynurenic acid levels, increased quinolinic acid concentrations, and elevated quinolinic acid/picolinic acid ratios in patients with PD (47, 48).

NAMPT is a rate-limiting enzyme in the NAD^+^ salvage pathway and plays a critical role in regulating cellular metabolism associated with aging, immune function, and neurodegenerative disorders. Previous studies have demonstrated that NAMPT exerts neuroprotective effects in the aging brain and in models of neurodegenerative diseases. Clinical studies have shown that NAMPT mRNA expression is significantly elevated in the peripheral blood of patients with PD (49). Notably, increased NAMPT expression in peripheral blood may reflect an adaptive compensatory response to systemic NAD^+^ depletion and metabolic stress, rather than an enhancement of NAMPT activity in the central nervous system. Animal studies further revealed that dopaminergic neurons in the substantia nigra of PD models exhibit higher NAMPT expression levels compared with other neuronal populations. Conditional deletion of the NAMPT gene in dopaminergic neurons, or administration of NAMPT inhibitors into the substantia nigra of mice, induces time-dependent loss of dopaminergic neurons, impairment of the nigrostriatal dopaminergic pathway, and progressive motor deficits (50). In a rotenone-induced PD model, both NAD^+^ and NAMPT levels were significantly reduced in the rat striatum (51). Similarly, in an *in vitro* PD model, treatment with the neurotoxin 6-hydroxydopamine (6-OHDA) markedly decreased intracellular NAMPT and NAD^+^ levels, accompanied by a significant reduction in the NAD^+^/NADH ratio. Pretreatment with the highly specific NAMPT inhibitor FK866 markedly exacerbated the inhibitory effects of 6-OHDA on PC12 cell viability, whereas supplementation with nicotinamide mononucleotide (NMN), the enzymatic product of NAMPT, exerted a protective effect. Further investigations demonstrated that NMN significantly reversed 6-OHDA-induced reductions in superoxide dismutase (SOD) activity and glutathione (GSH) levels, while suppressing the elevation of malondialdehyde (MDA) and lactate dehydrogenase (LDH) levels induced by 6-OHDA. In addition, 6-OHDA markedly reduced SIRT1 activity in PC12 cells, whereas NMN treatment effectively restored SIRT1 activity and attenuated this effect (52).

In addition to alterations in the NAMPT-mediated salvage pathway, impaired NAD^+^ biosynthesis through NMNAT enzymes may also contribute to NAD^+^ metabolic dysfunction in PD. NMNAT1 has been implicated in axonal protection and Wallerian degeneration. Previous studies demonstrated that NMNAT1 protects neurons and axons from degeneration in mouse and Drosophila models. In cultured cortical neurons, knockdown of NMNAT1 significantly impaired dendritic outgrowth, branching, and axonal development, whereas modulation of NMNAT1 expression altered neuronal morphology. These findings suggest that disruption of NAD^+^ biosynthetic capacity may contribute to neuronal structural vulnerability and neurodegenerative processes, providing additional mechanistic links between NAD^+^ metabolism and neuronal survival (53). A recent study demonstrated that NMNAT3 protein levels were significantly reduced in the caudate nucleus of patients with PD, and this reduction was inversely correlated with monomeric α-synuclein levels. In dopaminergic SH-SY5Y cells overexpressing α-synuclein, decreased NMNAT3 expression was accompanied by neurite pathology, whereas inhibition of NAMPT with FK866 partially rescued these abnormalities. These findings suggest that α-synuclein pathology may directly disrupt NAD^+^ biosynthetic capacity, thereby contributing to synaptic dysfunction and neurodegeneration in PD (54).

## 5 Preclinical evidence for NAD^+^ supplementation strategies

Direct restoration of NAD^+^ levels has also been demonstrated to exert potential neuroprotective effects in PD models. In a 6-OHDA-induced mouse model of PD, local administration of NAD^+^ into the striatum significantly ameliorated motor deficits and reduced dopaminergic neuronal damage in both the substantia nigra and striatum. Furthermore, in PC12 cell models, NAD^+^ pretreatment improved cell viability and alleviated 6-OHDA-induced oxidative stress and mitochondrial dysfunction, suggesting that NAD^+^ replenishment may exert anti-parkinsonian effects through the restoration of mitochondrial homeostasis (55). However, due to the pharmacokinetic limitations associated with direct NAD^+^ supplementation, recent studies have increasingly focused on the use of NAD^+^ precursors, such as NR and NMN, as more effective strategies to enhance intracellular NAD^+^ availability. Among these precursors, NR and NMN have emerged as promising NAD^+^-boosting compounds in recent years due to their favorable bioavailability and tissue distribution properties. Although NA is also an effective NAD^+^ precursor, its clinical application may be limited by adverse effects such as cutaneous flushing (Tables 1, 2).

**Table 1:** The clinical study.

Study	Intervention	Study design	Participants	Dose and duration	Major findings	Limitations and considerations
NADPARK trial (57)	NR	Randomized, double-blind, placebo-controlled phase I clinical trial	Patients with early-stage PD (*n* = 30)	NR supplementation for 30 days	NR significantly increased brain NAD^+^ levels and altered cerebral metabolic patterns. Patients with greater increases in brain NAD^+^ exhibited mild clinical improvement. NR also induced transcriptional changes associated with mitochondrial, lysosomal, and proteasomal functions.	The improvement in MDS-UPDRS scores was correlated with shorter intervals between the last levodopa dose and clinical assessment, suggesting that part of the clinical benefit may be attributed to a synergistic effect with levodopa. Considerable inter-individual variability in brain NAD^+^ elevation was observed.
NR-SAFE trial (56)	NR	Randomized, double-blind, placebo-controlled safety trial	Patients with PD (*n* = 10)	NR 3,000 mg/day for 4 weeks	High-dose NR was well tolerated and induced robust peripheral NAD^+^ metabolic responses, increasing blood NAD^+^ concentrations by approximately five-fold. Clinical improvement in MDS-UPDRS scores was also observed.	Small sample size and short follow-up duration limited evaluation of long-term efficacy. The clinical improvement may have been influenced by levodopa administration intervals.
N-DOSE trial (58)	NR	Randomized, double-blind, dose-escalation trial	Patients with PD (*n* = 29)	Multiple dose levels of NR	Designed to determine the optimal biological dose of NR and characterize dose-response relationships, providing essential information for future therapeutic trials.	Long-term disease-modifying effects remain unknown.

**Table 2:** The animal study.

References	Model system	Intervention	PD model	Major findings	Proposed mechanisms
Shan (55)	Mousel; PC12 cell	Direct NAD^+^ supplementation (intrastriatal NAD^+^ injection; NAD^+^ pretreatment in cells)	6-OHDA-induced PD mouse model; PC12 cell model	NAD^+^ administration ameliorated motor deficits and reduced dopaminergic neuronal damage in the substantia nigra and striatum. In PC12 cells, NAD^+^ pretreatment improved cell viability and alleviated 6-OHDA-induced morphological damage.	NAD^+^ exerted neuroprotective effects by reducing oxidative stress and improving mitochondrial dysfunction.
Luo (59)	Zebrafish	NR	MPTP-induced PD model	NR alleviated motor dysfunction, prolonged survival, and protected dopaminergic and peripheral neurons. Transcriptomic and metabolomic analyses revealed metabolic remodeling after NR treatment.	NR improved glucose metabolism and attenuated endoplasmic reticulum stress through activation of the PERK–eIF2α-ATF4–CHOP pathway.
Turconi (60)	Mouse	NR	Proteasome inhibitor-induced PD model	Short-term NR treatment partially improved behavioral deficits; however, prolonged NR administration reduced dopamine levels and downregulated dopamine-related genes (Drd2, Th, Maoa, and Maob).	Long-term NR exposure may suppress 26S/20S proteasome activity, increasing vulnerability of nigrostriatal dopaminergic neurons.
	*Caenorhabditis elegans*	NR	α-synuclein overexpression model	NR rescued α-synuclein-associated phenotypes, improved mitochondrial function and locomotor activity, and prevented age-related dopaminergic neuron loss.	Activation of mitochondrial unfolded protein response (UPR∧mt) and enhancement of mitochondrial homeostasis.
Schöndorf (61)	Patient-derived iPSC neurons	NR	GBA-associated PD model	NR increased intracellular NAD^+^ levels and significantly improved mitochondrial dysfunction in patient-derived dopaminergic neurons.	Restoration of mitochondrial metabolic homeostasis.
	*Drosophila melanogaster*	NR	GBA-associated PD genetic model	NR prevented age-dependent dopaminergic neuron loss and motor decline.	Improvement of mitochondrial function and NAD^+^ metabolic balance.
Zhou (63)	Cellular models and mouse model	NMN (NAD^+^ booster); CD38 knockdown (siCD38); nicotinamide adenine dinucleotide translocator-related intervention (NAT)	Rotenone-induced cellular PD model and MPTP-induced PD mouse model	NAD^+^ augmentation strategies increased mitochondrial quality control (MQC), reduced pro-inflammatory cytokine expression, inhibited apoptosis, and enhanced redox homeostasis in rotenone-treated cells. In MPTP-induced PD mice, NMN supplementation alleviated motor deficits and prevented PD-associated neuropathological alterations.	Restoration of intracellular NAD^+^ levels enhanced mitochondrial unfolded protein response (UPR∧mt) and mitophagy. The protective effects of NMN were dependent on the ATF4-mediated mitochondrial UPR pathway. Multi-omics and metabolomic analyses revealed NMN-induced transcriptional and metabolic remodeling associated with mitochondrial homeostasis in the striatum
Lu (64)	PC12 cells	NMN	Rotenone-induced cellular PD model	NMN increased cell viability, restored intracellular NAD^+^ and ATP levels, reduced oxidative damage markers, and attenuated apoptosis.	Improvement of mitochondrial energy metabolism, reduction of oxidative stress, and restoration of SIRT1 activity.
Yu (66)	Rat	NMNAT2 supplementation	6-OHDA-induced PD model	NMNAT2 supplementation alleviated neuronal injury, motor impairment, and oxidative stress.	Restoration of NAD^+^ biosynthetic capacity and maintenance of neuronal mitochondrial homeostasis.
Hu (69)	SH-SY5Y cells, *Caenorhabditis elegans*, and mice	NAD^+^ supplementation combined with PARP1 inhibitor PJ34	6-OHDA-induced PD models	Combined NAD^+^ supplementation and PJ34 treatment enhanced neuroprotective effects compared with either intervention alone. The combination reduced oxidative stress, preserved mitochondrial integrity, suppressed cellular senescence, promoted autophagy, and alleviated neuroinflammation across cellular, nematode, and mouse models.	PJ34 inhibited PARP1 activation and prevented excessive NAD^+^ consumption and DNA damage signaling, whereas NAD^+^ replenishment restored mitochondrial and metabolic homeostasis through SIRT1 and AMPK pathways. The synergistic effect suggests complementary regulation of NAD^+^ metabolism, mitochondrial function, and stress responses.
Lehmann (70)	Drosophila melanogaster	Nicotinamide (NAM) supplementation; *Parp* mutation	*pink1* mutant PD model	*pink1* mutant flies exhibited altered NAD^+^ salvage metabolism and mitochondrial defects. Dietary supplementation with nicotinamide rescued mitochondrial dysfunction and protected dopaminergic neurons from degeneration. Loss of *Parp* also improved mitochondrial function and reduced neuronal toxicity.	Increasing NAD^+^ availability through precursor supplementation or reducing NAD^+^ consumption by PARP inhibition restored NAD^+^ homeostasis, alleviated mitochondrial dysfunction, and protected neurons from degeneration.
Roshdy (67)	rats	Nicotinic acid (NIA) supplementation	Rotenone-induced PD rats	Improved motor deficits, increased TH expression, preserved dopaminergic neurons in SNpc and striatum	ctivated PKC/LKB1/AMPK signaling, suppressed ER stress (ATF4/ATF6/XBP1s) and apoptosis (CHOP/JNK/caspase-3)
Saha (62)	mouse	NR	Double-hit PD mouse model (A53T α-synuclein overexpression combined with chronic benomyl exposure)	NR significantly improved motor performance and enhanced striatal dopamine neurotransmission. However, NR did not prevent nigral dopaminergic neuron degeneration.	NR increased ATP/ADP ratios in substantia nigra dopaminergic neurons and striatal spiny projection neurons, indicating enhanced mitochondrial bioenergetic capacity. The beneficial effects were attributed to improved neuronal energy metabolism and dopamine release rather than direct preservation of dopaminergic neurons.
Rehman (65)	mouse	NAM	MPTP-induced PD mouse model	NAM improved motor deficits and reversed PD-associated changes, including reduced α-synuclein accumulation and restoration of TH and DAT expression in the substantia nigra and striatum.	NAM enhanced antioxidant defense via activation of the Nrf2/HO-1 pathway and suppressed neuroinflammation through inhibition of TLR4/NF-κB signaling.
Altay (68)	rats	Combined metabolic activators (CMA1: nicotinamide riboside + L-serine + N-acetyl cysteine + L-carnitine tartrate; CMA2: nicotinamide + L-serine + N-acetyl cysteine + L-carnitine tartrate)	Rat PD model	Both CMA1 and CMA2 improved neuronal pathological changes, including reduced neuronal hyperemia, degeneration, and necrosis. CMA2 showed superior effects compared with CMA1. In PD rat models, both formulations improved behavioral performance and metabolic parameters.	CMA treatment may enhance mitochondrial metabolism, restore cellular energy homeostasis, increase antioxidant capacity through glutathione precursors, and improve NAD^+^ metabolism through NR/NAM supplementation.

### 5.1 NR

Clinical studies have provided preliminary evidence supporting the therapeutic potential of NR supplementation in patients with PD. NR intervention was reported to significantly improve the total score of the Movement Disorder Society-Unified Parkinson's Disease Rating Scale (MDS-UPDRS). The NR-SAFE trial further demonstrated that the highest tested dose of NR (3,000 mg/day) was well tolerated and capable of inducing robust peripheral metabolic responses. Although this study observed an improvement in clinical disease severity, it should be noted that the magnitude of improvement was associated with a shorter interval between the last levodopa administration and clinical assessment, suggesting that the observed benefits may have been partially influenced by enhanced levodopa responsiveness. In addition, NR supplementation markedly increased systemic NAD^+^ metabolism, resulting in an approximately five-fold elevation in blood NAD^+^ levels. Although a mild increase in serum homocysteine was observed during the early intervention period, the overall integrity of the methyl donor pool remained preserved (56).

Given that NR metabolism increases the production of NAM, which undergoes methylation-dependent degradation and may potentially influence one-carbon metabolism and DNA methylation homeostasis, concerns have been raised regarding the long-term metabolic safety of NR supplementation. A recent randomized, double-blind, placebo-controlled clinical trial involving 29 patients with PD investigated the effects of short-term high-dose NR administration (30 days) on DNA methylation homeostasis. The study demonstrated that NR supplementation did not induce significant alterations in global DNA methylation patterns in peripheral blood cells or skeletal muscle. Importantly, no adverse effects on methylation homeostasis were observed even in individuals carrying pathogenic MTHFR variants associated with altered one-carbon metabolism. These findings suggest that short-term high-dose NR supplementation has a favorable epigenetic safety profile; however, the long-term effects of NR on methyl metabolism and epigenetic regulation require further investigation (57).

Beyond symptomatic improvement, NR supplementation induces multidimensional metabolic remodeling in patients with PD. Using phosphorus-31 magnetic resonance spectroscopy (∧31P-MRS), researchers found that NR treatment significantly increased brain NAD^+^ levels, although considerable interindividual variability was observed. Corresponding alterations in NAD^+^-related metabolites were also detected in cerebrospinal fluid. Furthermore, ∧18F-fluorodeoxyglucose positron emission tomography (∧18F-FDG PET) revealed that patients exhibiting increased brain NAD^+^ levels after NR treatment showed altered cerebral metabolic patterns, and these metabolic changes were associated with modest improvements in clinical symptoms. In addition, NR significantly improved the NAD^+^ metabolomic profiles of circulating blood cells and skeletal muscle and upregulated transcriptional programs related to mitochondrial, lysosomal, and proteasomal functions. Concurrently, inflammatory cytokine levels in both serum and cerebrospinal fluid were significantly reduced following NR intervention (58).

In a zebrafish model of PD induced by MPTP, NR ameliorated motor deficits, prolonged survival, protected dopaminergic neurons and peripheral neurons, and increased systemic NAD^+^ levels. Transcriptomic analysis revealed that differentially expressed genes following NR treatment were significantly enriched in glucose metabolism pathways and endoplasmic reticulum (ER) protein processing pathways. Quantitative PCR validation demonstrated that NR treatment significantly reduced the mRNA expression levels of gluconeogenesis-related enzymes while increasing the expression of glycolysis-associated enzymes. Untargeted metabolomic analysis further showed that NR supplementation markedly increased the levels of nicotinic acid, nicotinamide, D-glucose, and gluconeogenic amino acids such as glutamine in model fish. Importantly, NR alleviated MPTP-induced endoplasmic reticulum stress (ERS) through activation of the PERK–eIF2α-ATF4–CHOP signaling pathway (59). In Caenorhabditis elegans, NR activates the mitochondrial unfolded protein response and rescues phenotypes induced by α-synuclein overexpression. NR also enhances mitochondrial function and locomotor activity and prevents age-related dopaminergic neuronal loss in α-synuclein-overexpressing models (60). In a rotenone-induced mouse model of PD, NR treatment rescued dopaminergic neuron loss, improved nigrostriatal dopaminergic pathway impairment, and alleviated Parkinsonian motor deficits (50).

However, the effects of NR are not consistent across different PD models. In a proteasome inhibitor-induced PD mouse model, NR initially partially rescued behavioral deficits, whereas long-term treatment reduced dopamine levels and the expression of dopamine-related genes, including *Drd2, Th, Maoa*, and *Maob*, in the substantia nigra. The authors suggested that prolonged NR treatment may suppress 26S/20S proteasome activity, thereby potentially increasing the risk of nigrostriatal dopaminergic dysfunction (60). Furthermore, studies focusing on GBA-associated Parkinson's disease (GBA-PD), one of the most common genetic risk forms of PD, have utilized patient-derived induced pluripotent stem cells (iPSCs) and Drosophila melanogaster models. Patient-derived neurons exhibited enhanced stress responses, mitochondrial impairment, and altered NAD^+^ metabolism. NR supplementation increased NAD^+^ levels and significantly improved mitochondrial function in these patient-derived neurons. Similarly, in GBA-PD fly models, NR prevented age-related dopaminergic neuron loss and locomotor decline (61). Taken together, current evidence indicates that the neuroprotective effects of NR in PD are highly heterogeneous and dependent on experimental conditions. Factors including intervention timing, dosage, and disease model type (toxin-induced, genetic, or proteasome inhibition-based models) may substantially influence therapeutic outcomes. Therefore, careful optimization of treatment strategies will be essential before clinical application of NR-based interventions. Furthermore, recent studies have suggested that the beneficial effects of NR supplementation in PD may not solely depend on preventing dopaminergic neuronal loss but may also involve the improvement of neuronal metabolic function and synaptic neurotransmission. In a “double-hit” PD mouse model combining A53T α-synuclein overexpression in substantia nigra dopaminergic neurons with chronic benomyl exposure, continuous NR supplementation significantly improved motor performance. However, unlike conventional PD models, NR treatment did not prevent the degeneration of nigral dopaminergic neurons. Instead, NR enhanced striatal dopamine neurotransmission and improved neuronal bioenergetic status. Further analyses demonstrated that NR increased the ATP/ADP ratio in both substantia nigra dopaminergic neurons and striatal spiny projection neurons, indicating enhanced mitochondrial energy capacity. Moreover, NR promoted both tonic and stimulus-evoked dopamine release in the striatum. These findings suggest that NAD^+^ augmentation may alleviate PD-related functional deficits through metabolic and functional modulation, rather than exclusively through the preservation of dopaminergic neuron survival, providing new insights into the mechanisms underlying NAD^+^-based therapeutic strategies for PD (62).

### 5.2 NMN

Among the currently investigated NAD^+^ precursors, NMN has emerged as a highly promising therapeutic candidate due to its high bioavailability and extremely low toxicity profile (63). *In vivo* studies have demonstrated that NMN supplementation alleviates motor deficits and prevents neuropathological alterations in MPTP-induced PD mouse models. Mechanistic investigations suggest that these protective effects are closely associated with activation of the ATF4-mediated mitochondrial unfolded protein response (UPR∧mt) pathway. Multi-omics and metabolomic analyses of the striatum revealed that NMN induces transcriptional alterations in genes and proteins associated with mitochondrial homeostasis. The accelerated pathological progression observed in PD models is likely attributable, at least in part, to impaired mitochondrial quality control (MQC). Increasing intracellular NAD^+^ availability can alleviate mitochondrial proteotoxic stress, thereby mitigating PD-associated phenotypes. Further evidence indicates that NAD^+^ supplementation improves PD-related mitochondrial proteotoxicity, enhances striatal synaptic integrity, and reduces glial activation and neurodegeneration. Moreover, NMN treatment restores MQC and cellular homeostasis in the brains of PD mice through activation of UPR and mitophagy-related signaling pathways (63). In another study, a cellular PD model was established using rotenone-treated PC12 cells. MTT and lactate dehydrogenase (LDH) assays demonstrated that NMN significantly increased PC12 cell viability and restored intracellular NAD^+^ and ATP levels. These findings indicate that NMN can alleviate energy metabolic deficits caused by mitochondrial dysfunction, suggesting that NMN exerts beneficial effects in PD cellular models by attenuating apoptosis and improving mitochondrial energy metabolism (64).

### 5.3 Nicotinamide (NAM)

NAM, an amide form of vitamin B3, is a classical precursor in the NAD^+^ salvage pathway and can be converted into NMN by NAMPT. Although recent studies have focused mainly on NR and NMN supplementation, emerging evidence suggests that NAM may also exert neuroprotective effects in PD models. In an MPTP-induced mouse model of PD, NAM administration significantly alleviated motor deficits and reversed PD-associated pathological alterations, including increased α-synuclein accumulation and reduced tyrosine hydroxylase (TH) and dopamine transporter (DAT) expression in the substantia nigra and striatum. Mechanistically, NAM treatment enhanced antioxidant defense by activating the Nrf2/HO-1 pathway and attenuated neuroinflammation through suppression of TLR4/NF-κB signaling. These findings indicate that NAM supplementation may protect dopaminergic neurons by improving redox homeostasis and inflammatory responses, supporting the therapeutic potential of targeting NAD^+^ metabolism in PD (65).

### 5.4 NMNAT

NMNAT2 is one of the isoenzymes of the NMNAT family and catalyzes the critical conversion of NMN to NAD^+^. Recent studies have identified NMNAT2 as an essential factor for maintaining neuronal function. Its expression level is positively associated with cognitive performance and negatively correlated with pathological features of neurodegenerative diseases. In PD, NMNAT2 expression is significantly reduced and contributes to mitochondrial dysfunction, thereby influencing disease progression. Mechanistic studies have demonstrated that gut microbiota dysbiosis can exacerbate neurobehavioral deficits and oxidative stress in 6-OHDA-induced PD rat models by suppressing NMNAT2 expression, thereby promoting PD progression. Conversely, exogenous NMNAT2 supplementation effectively alleviates neuronal injury, motor impairment, and oxidative stress (66).

### 5.5 NIA

In addition to NR and NMN, nicotinic acid (NIA), a classical vitamin B3 derivative and NAD^+^ precursor, has also demonstrated potential neuroprotective effects in PD models. In a rotenone-induced rat model of PD, NIA treatment improved motor deficits and attenuated dopaminergic neuronal degeneration in the substantia nigra–striatal pathway. Mechanistically, NIA activated the PKC/LKB1/AMPK signaling cascade, thereby suppressing endoplasmic reticulum stress responses, as evidenced by reduced expression of ATF4, ATF6, and XBP1s, followed by attenuation of CHOP/GADD153, phosphorylated JNK, and active caspase-3-mediated apoptosis. Furthermore, NIA increased tyrosine hydroxylase immunoreactivity and preserved neuronal integrity in the substantia nigra and striatum. The reversal of these effects by the PKC inhibitor tamoxifen further suggests that PKC activation represents a critical mediator of NIA-induced neuroprotection (67).

### 5.6 Combination NAD^+^-boosting supplementation strategies

In addition to supplementation with individual NAD^+^ precursors or activators, researchers have developed combination metabolic activators designed to simultaneously target multiple metabolic pathways. A single-dose combination metabolic activator formulation consists of L-serine, N-acetyl-L-cysteine, NR, and L-carnitine tartrate. Clinical findings showed that although daily administration of the combination metabolic activator for the initial 28 days did not significantly improve motor function, PD patients receiving the combination intervention exhibited significant improvements in cognitive performance. Furthermore, among patients with advanced PD, treatment with the combination metabolic activator was associated with significantly improved MoCA scores, suggesting potential benefits for cognitive function. Functional magnetic resonance imaging (fMRI) analysis revealed that cognitive changes were associated with alterations in key brain networks, particularly regions involved in cognitive processing. Two formulations, namely CMA1 (NR, L-serine, N-acetyl-L-cysteine, and L-carnitine tartrate) and CMA2 (nicotinamide, L-serine, N-acetyl-L-cysteine, and L-carnitine tartrate), as well as their individual components, have been evaluated in animal models of AD and PD. Histological analyses demonstrated that both CMA1 and CMA2 improved neuronal pathological changes, including congestion, degeneration, and necrosis, in AD and PD models. Notably, CMA2 exhibited greater therapeutic efficacy than CMA1. Immunohistochemical analysis further revealed reduced neuronal immunoreactivity following treatment. In addition, both formulations enhanced metabolic activity in hepatic tissues. In PD rat models, administration of either formulation positively affected behavioral performance and significantly improved metabolic and motor functions, consistent with the observed neurohistological improvements (68).

### 5.7 Others

In addition to direct NAD^+^ precursor supplementation, recent studies have explored strategies aimed at preventing excessive NAD^+^ depletion. For example, combined NAD^+^ supplementation with PARP1 inhibition has shown synergistic neuroprotective effects in PD models by simultaneously restoring NAD^+^ availability and reducing NAD^+^-consuming stress responses (69). Early genetic studies have demonstrated that increasing NAD^+^ availability can protect against PD-associated mitochondrial defects. In *Drosophila pink1* mutants, nicotinamide supplementation restored NAD^+^ salvage metabolism, improved mitochondrial function, and prevented neuronal degeneration. Similarly, genetic inhibition of PARP reduced NAD^+^ consumption and exerted neuroprotective effects, suggesting that limiting NAD^+^ depletion represents an alternative therapeutic strategy for PD (70).

The clinical translation of NAD^+^-based therapies still faces several critical challenges. First, substantial inter-individual variability remains a major concern. The NADPARK trial revealed marked heterogeneity in responses to NR supplementation among participants, with some individuals exhibiting pronounced increases in brain NAD^+^ levels, whereas others showed limited changes. Such variability may be attributed to multiple factors, including genetic background, baseline nutritional status, and disease stage. The dose-escalation design of the N-DOSE trial was specifically developed to investigate strategies for optimizing individual responses to NAD^+^-boosting interventions. Second, the dose–response relationship of NAD^+^ supplementation remains unclear. NAD^+^-boosting therapies may exhibit nonlinear dose-dependent effects. Although higher doses may theoretically provide greater therapeutic benefits, excessive NR or nicotinamide supplementation could increase methylation demand due to enhanced nicotinamide methylation, thereby imposing additional metabolic stress on methyl donor availability and potentially affecting neurotransmitter biosynthesis. Further clinical studies, including the N-DOSE trial, are required to systematically evaluate the effects of different dosing regimens on methyl metabolism and neurotransmitter homeostasis. Third, long-term safety remains an important consideration. Currently completed clinical trials have relatively short follow-up durations (approximately 4–12 weeks), whereas PD is a chronic and progressive neurodegenerative disorder requiring long-term intervention. The 78-week follow-up period and open-label extension phase of the NADAPT trial are expected to provide valuable evidence regarding the long-term safety profile of NAD^+^-boosting therapies. Finally, the extent of NAD^+^ depletion may vary according to PD subtype, disease duration, and genetic background. Future studies should focus on developing reliable biomarkers to identify patient populations most likely to benefit from NAD^+^ supplementation, thereby enabling a more personalized and precision medicine-based therapeutic approach.

## 6 Discussion

NAD^+^ metabolism appears to occupy an important position in the pathophysiology of PD, linking aging, mitochondrial dysfunction, impaired proteostasis, DNA damage, and neuroinflammation. Overall, the available evidence provides relatively consistent support for an association between NAD^+^ depletion and neuronal vulnerability. Preclinical studies further indicate that restoring NAD^+^ levels with precursors such as NR and NMN can improve mitochondrial function, enhance mitochondrial quality control, reduce oxidative stress, and protect dopaminergic neurons in several experimental models. These findings support NAD^+^ metabolism as a promising therapeutic target. However, the strength of the evidence varies substantially across experimental and clinical settings.

Importantly, the effects of NAD^+^ augmentation are not uniformly beneficial. Although NR has demonstrated neuroprotective effects in several PD models, including improvements in mitochondrial function and dopaminergic neuronal survival, prolonged NR treatment has also been reported to suppress 26S/20S proteasome activity and worsen dopaminergic outcomes in a proteasome inhibitor-induced model. Such divergent findings suggest that the biological effects of NAD^+^ restoration may be context-dependent and influenced by dose, treatment duration, disease stage, experimental model, and the pre-existing state of mitochondrial and proteostasis networks. Thus, the current evidence does not support a simple “more NAD^+^ is better” model. Instead, an appropriate level and duration of NAD^+^ augmentation may be required to achieve therapeutic benefit while avoiding disruption of other metabolic pathways.

The clinical evidence should also be interpreted cautiously. Early trials have demonstrated that NR can substantially increase systemic NAD^+^ availability and alter NAD^+^-related metabolic profiles, with some studies reporting improvements in clinical measures. However, improvements in MDS-UPDRS scores were associated with a shorter interval between the last levodopa administration and clinical assessment, raising the possibility that part of the observed symptomatic benefit may be related to levodopa rather than a direct disease-modifying effect of NR. Therefore, current clinical studies provide encouraging evidence of target engagement and potential symptomatic benefit, but remain insufficient to establish whether NAD^+^ augmentation can slow or modify the underlying progression of PD. Longer and adequately controlled trials with standardized levodopa assessment conditions are required to address this question.

Several important gaps therefore remain. First, future studies should clarify the cell-type-specific effects of NAD^+^ metabolism in dopaminergic neurons, microglia, and astrocytes. Second, biomarkers reflecting tissue NAD^+^ status and treatment responsiveness are needed to identify patients most likely to benefit from NAD^+^ augmentation. Third, the interactions between NAD^+^ supplementation and established PD therapies, particularly levodopa, should be systematically evaluated. Fourth, dose optimization and long-term safety require further investigation, given the heterogeneous responses observed across models and individuals. Finally, integrating metabolomic, transcriptomic, and genetic data may help explain interindividual variability and determine whether NAD^+^ restoration can ultimately exert disease-modifying effects rather than merely improve symptoms.

## Data Availability

The original contributions presented in the study are included in the article/supplementary material, further inquiries can be directed to the corresponding author.
